# Sleep inadequacy and the relationship with mucosal immunity and upper respiratory symptoms in elite swimmers: A longitudinal study leading into the Commonwealth Games

**DOI:** 10.1371/journal.pone.0346138

**Published:** 2026-04-02

**Authors:** Lauren H. Baker, Terun Desai, Jonathan Sinclair, Amy V. Wells

**Affiliations:** 1 Centre for Research in Psychology and Sports, School of Health, Medicine and Life Sciences, University of Hertfordshire, Hertfordshire, United Kingdom; 2 Division of Surgery & Interventional Science, Institute of Sport, Exercise & Health, University College London, London, United Kingdom; 3 Research Centre for Applied Sport, Physical Activity and Performance, School of Sport & Health Sciences, Faculty of Allied Health and Wellbeing, University of Central Lancashire, Lancashire, United Kingdom; Murdoch University, AUSTRALIA

## Abstract

**Objectives:**

To monitor sleep patterns of elite swimmers and explore sleep as a potential risk factor for upper respiratory symptoms (URS) alongside salivary Immunoglobulin A (IgA) in elite swimmers, over an 8-month competitive season.

**Design:**

Secondary analysis of an 8-month longitudinal study in elite international swimmers leading into either the Commonwealth Games 2018 or Swim Cup Eindhoven.

**Methods:**

Fourteen elite swimmers (age ± SD = 19.9 ± 0.8 years, height = 178.9 ± 6.3 cm, and mass = 75.0 ± 7.7 kg) were recruited. Self-reported sleep quality, URS data and salivary IgA was obtained weekly on a standardised day. Quantitative sleep parameters were measured using wrist-worn actigraphy four times for two-week bouts; during low, moderate, high training loads and once leading into competition.

**Results:**

Swimmers waking fatigued was positively associated with frequency (p < 0.001) and severity (p < 0.001) of URS, plus negatively associated with salivary IgA (p = 0.035). Perception of meeting 7–9 hour national sleep recommendations was positively associated with URS frequency (p < 0.001) and severity (p = 0.001). Average sleep duration was 06:30 hrs: mins and reduced significantly during high training loads (p = 0.001) and early morning training (5:00 hrs: mins, p = 0.001). Average sleep efficiency was 81% over the 8-month period.

**Conclusions:**

Perceived fatigue on waking was significantly associated with both frequency and severity of URS, and inversely associated with mucosal immunity (salivary IgA), providing novel insight into the relationship between sleep, fatigue and illness in this cohort. Although causality cannot be established, the high prevalence of inadequate sleep shown in elite swimmers highlights the importance of individual sleep monitoring to support recovery and inform strategies aimed at illness prevention.

## Introduction

Inadequate sleep can impair cognitive function, mood and the physiological processes required for recovery and adaption [1], subsequently, affecting athletic performance [2]. Poor sleep can also decrease immune function [2]; associations have been seen between short sleep duration (< 7 hours) and increased number of illnesses [3,4]. Despite knowing the importance of sleep, restricted sleep quantity has been continually reported amongst elite athletes (n = 98) from a multitude of different sports [5]. One study analysing 926 sleep periods [6], reported that 60% of athletes were not able to achieve the national recommendation (NR) [7] of seven to nine hours total sleep time; which was recently supported by a systematic review and meta-analysis of 54 studies [1]. More specifically, previous research has shown a lack of sleep in swimmers [6,8,9], with one study reporting 06:14 hrs: mins total sleep time (TST) (n = 108) [10]. Interestingly, the level of athlete had no influence, as a study in Olympic swimmers showed similar findings [11]. Sleep efficiency, a key measure of sleep quality (calculated by total sleep time, divided by total time in bed), has been recommended to be a minimum of 85% [12]. However, evidence has continually showed poorer sleep quality in athletes, than a non-athletic population [8,10,13]. In elite swimmers specifically, sleep efficiency of 82–84% has been reported [11,14], below the identified healthy threshold [12]. Considering this, plus the significant impact that inadequate sleep quantity specifically could have on immune function; findings provide strong rationale to develop a deeper understanding of the relationship between sleep and illness in elite swimmers.

Overall, there is no clear consensus on whether a causal relationship exists between athletic training and sleep adequacy [15]. However, sport specific risk factors known to impair sleep include high training loads [16], early morning training [9], and competition [17,18]. Few published studies have examined the effect of training load for swimmers on sleep, thus presenting a significant gap within the research. However, swimming studies have reported an average sleep duration of 05:24 hrs: mins on nights prior to early morning training [9,10]. Plus, retiring to bed earlier the night before training did not seem to significantly help the reduction in sleep duration [9]. Poor sleep has also been reported leading into competition; out of 632 athletes, 65.8% reported experiencing worse than normal sleep on the night before competition [18]. Similarly, 64% of athletes experienced the same before competition in another large cohort of 283 elite Australian athletes [17]. Taken with the fact that athletes rated sleep as vital for successful performance and that poor sleep quality has shown to decrease the odds of winning [19]; there is a growing urgency to understand sleep patterns of elite swimmers for early morning training, over differing training loads and leading into competition [6,9].

Our previous work highlighted the need for an individualised approach to athlete monitoring [20]. It was proposed that this could help identify those at risk of frequent upper respiratory symptoms (URS) and to explore other potential risk factors associated. One limitation of this work was that other factors that play an important role in this complex relationship, such as sleep, was not addressed [20]. Thus, it was deemed important to analyse both self-reported and objective sleep in conjunction with URS and salivary Immunoglobulin A (IgA) [20], over differing training loads and leading into competition. Therefore, the aims of this secondary analysis was to identify whether sleep was a potential risk factor of URS and changes in salivary IgA in elite swimmers over a competitive season. It was hypothesised that a relationship would exist between sleep and URS. The rarity of longitudinal access to elite swimmers makes this especially valuable, adding novel findings to the limited availability of studies.

## Methods

This study uses data collected concurrently with the original [20] but focuses on outcomes not reported in the initial publication due to word limits. As such, this represents a secondary analysis of the original dataset. A total of 23 elite swimmers were recruited between 21/08/2017 and 31/09/2017; however, nine did not complete the eight-month observation due to retiring during the study (n = 4) or because they did not train on the chosen analysis day due to university commitments (n = 5). Therefore, 14 elite national and international swimmers were included (age ± SD = 19.9 ± 0.8 years, height = 178.9 ± 6.3 cm, mass = 75.0 ± 7.7 kg, male = 64%). Swimmers were within two training groups: sprint (43%) and middle-distance/distance (57%). A total of 10 swim sessions a week were programmed: five early morning sessions and five afternoon/evening sessions (plus S&C sessions). Number of sessions to be attended by swimmers was individualised by coach decision, training group and competition schedule. Prior to study commencement, swimmers provided written fully informed consent and health screens. Ethical approval was granted for human investigation by The University of Hertfordshire, Health Science Engineering & Technology ECDA (Ethics protocol number: aLMS/PGR/UH/02940(1,2,3)). Patients and the public were not involved in design, conduct or reporting of this research, in any way.

**Fig 1:**
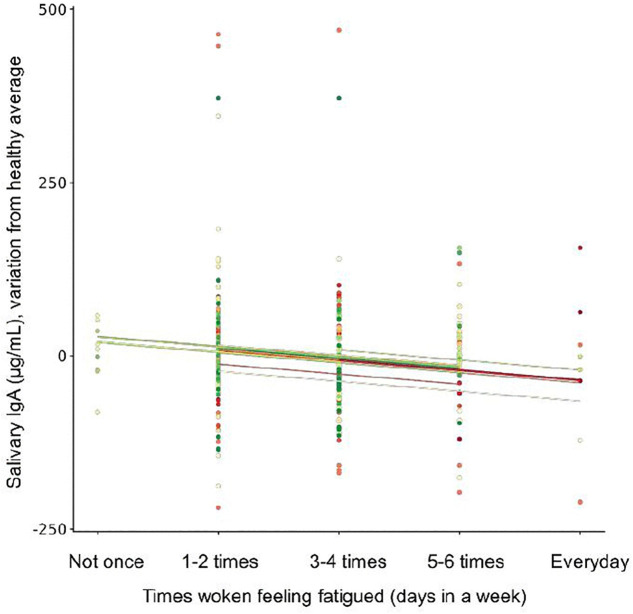
Study design flow diagram.

A study design flow diagram is shown in Fig 1 and outlines methodology used, which has been formerly outlined [20]. Self-reported sleep data was obtained alongside URS data weekly using an adaption of the Australian Institute of Sport (AIS) monthly illness log (S1 File) [21]. Self-perceived sleep quality was monitored by ranking on a scale of 1–10 (1 = poor, 10 = excellent) each week. Moreover, swimmers were asked how many times they thought they had met the NR of 7–9 hours and how many times they had awoken feeling fatigued each week. This sleep data was compared to relative salivary IgA (normalised to each individual’s healthy mean), URS, and coach derived training loads of which the methodology has been previously defined [20].

To assess sleep-wake patterns, a wrist-worn activity monitor GT3X+ (ActiGraph, Florida, USA) was worn around the non-dominant wrist during night-time sleep and napping periods only. This was removed when swimmers arose from bed in the morning, or after napping, and swimmers completed daily sleep diaries, which were used to assist in identifying bedtime and wake time for later analysis. Parameters recorded included latency (mins), sleep efficiency (SE; %), total time in bed (TTIB; min), total sleep time (TST; min), wake after sleep onset (WASO; min), number of awakenings, and average time awake (min). Actigraphy data was used to determine objective sleep-wake patterns for different training intensity periods (low, moderate, high training loads and into competition). Actigraphy data was processed using ActiLife software (version 6.13) and individual sleep data reports created by this software were provided to swimmers at the cessation of the study.

Data was compiled using Microsoft Excel and evaluated using both SPSS software (version 26.0; SPSS Lead Technologies Inc, Chicago, IL) and GraphPad Prism 8.0 (GraphPad Software, Inc., San Diego, CA). Results were presented as mean ± SD, and significance was accepted at the p ≤ 0.05 level. Repeated measures correlations were used to determine relationships between self-reported sleep parameters, URS and salivary IgA. Data was reported as *r*_*rm*_ and its 95% confidence interval (95% CI). The strength of the relationship was determined using the standard correlation coefficient interpretation scale used elsewhere [22] classified as no relationship (0), weak (0.1–0.3), moderate (0.4–0.6), strong (0.7–0.9), and perfect (1.0). Repeated measures analysis of variance (ANOVA) with post-hoc Bonferroni adjustment measured differences in measures of URS and sleep parameters between training loads. Greenhouse-Geisser correction was applied upon violation of Mauchly’s test of sphericity for ANOVAs (p < .05). For ANOVA analyses, Partial Eta-Squared ($\overset{\text{2}}{\underset{partial}{\eta }}$) was used to report effect sizes which were classified as small (0.01–0.08), moderate (0.09–0.25) and large (>0.25).

## Results

**Fig 2:**
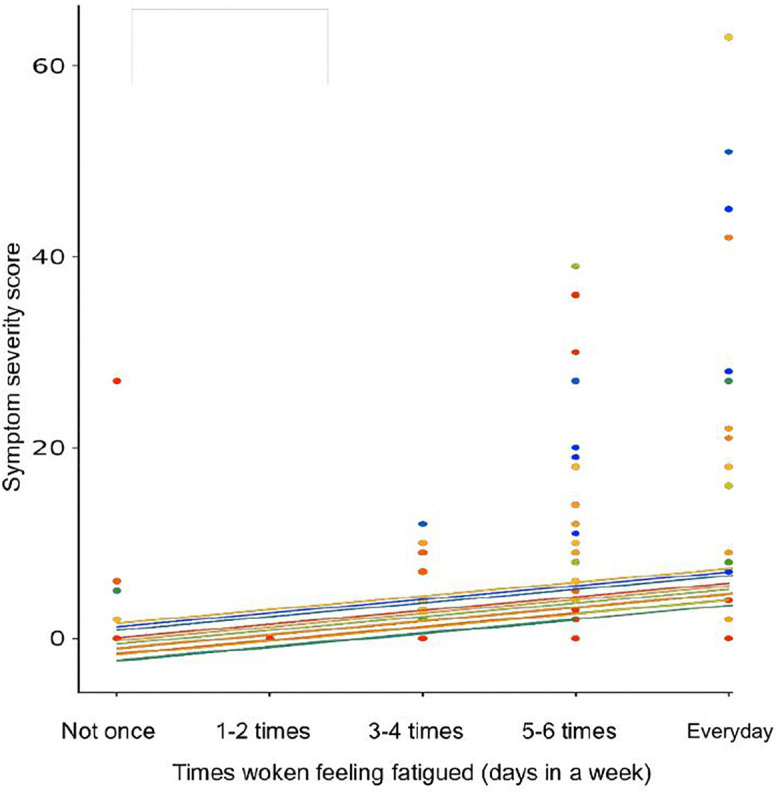
Reported times woken feeling fatigued alongside URS severity. A weak positive relationship was found between fatigue and symptom severity score (rrm (391) = 0.25, 95% CI [0.15, 0.336], p < 0.001) for the elite swimmers.

**Fig 3:**
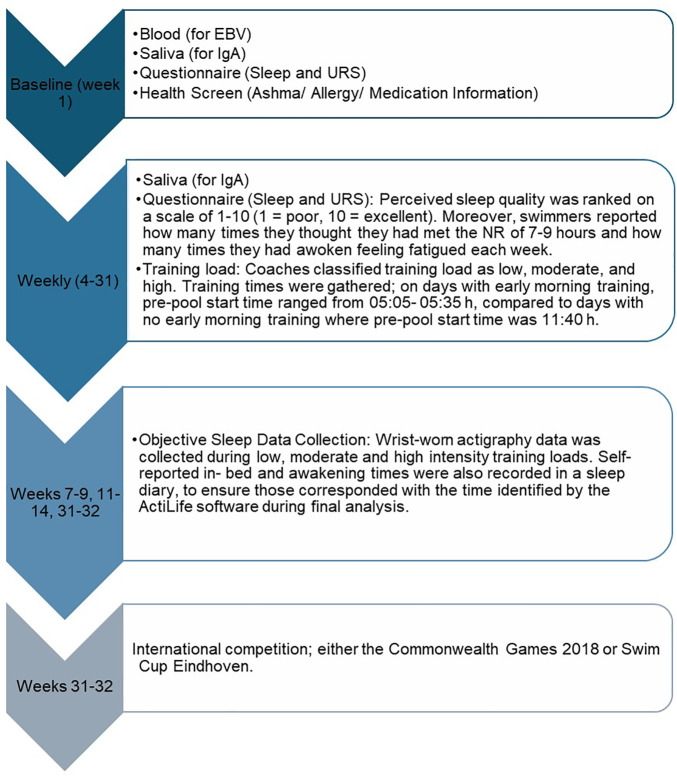
Reported times woken feeling fatigued alongside salivary IgA. A weak negative relationship was found with swimmers waking fatigued and relative salivary IgA (the variation from their healthy average) (rrm (304) = −0.12, 95% CI [−0.229, −0.008], p = 0.035).

A weak positive relationship was found between swimmers waking feeling fatigued with number of URS episodes (*r*_*rm*_ (391) = 0.27, 95% CI [0.176, 0.359], p < 0.001). Moreover, relationships were found between fatigue and URS severity (Fig 2) and fatigue and relative salivary IgA (Fig 3). A weak positive relationship was found between times the swimmers perceived they had met the NR of sleep with both number of URS episodes (*r*_*rm*_ (391) = 0.20, 95% CI [0.105, 0.295], p < 0.001) and symptom severity score (*r*_*rm*_ (391) = 0.16, 95% CI [0.065, 0.258], p = 0.001). However, no relationship was found between meeting the NR of sleep and relative salivary IgA (p = 0.281). Self-reported sleep score had no relationship with relative salivary IgA (p = 0.331), number of URS (p = 0.396) or URS severity (p = 0.416).

Over the eight-month study period, swimmers (n = 14) reported an average sleep quality score of 6.7 (out of 10). No significant differences were found for average sleep score between training loads (p = 0.570, $\overset{\text{2}}{\underset{partial}{\eta }}$ = 0.042) (low, 6.8 ± 1.0; moderate, 6.5 ± 1.1; high 6.5 ± 1.0). In total, 43% of swimmers reported sleeping the NR of 7–9 hours per night, 5–6 times a week. This was followed by 3–4 times a week (24%), everyday (23%), 1–2 times (9%) and not once (1%), respectively. When compared to ActiGraph data, swimmers met the NR on average 45% of the time, over the whole study period (range: 14–80%, median: 48%). A significant interaction was found for meeting NR 5–6 days (p = 0.001, $\overset{\text{2}}{\underset{partial}{\eta }}$ = 0.457); perceived more during moderate (p = 0.014) and high (p = 0.001) training loads, than low. Another interaction was found for 3–4 days (p = 0.001, $\overset{\text{2}}{\underset{partial}{\eta }}$ = 0.410); reported significantly more during high (p = 0.004), than during low training load weeks. Additionally, results showed 41% of swimmers reported waking feeling fatigued, 1–2 times a week. This was followed by 3–4 times (35%), 5–6 times (18%), not once (4%) and every day (3%), respectively. Swimmers woke feeling fatigued 3–4 days per week significantly more during high training loads, compared to low (*p* = 0.001, $\overset{\text{2}}{\underset{partial}{\eta }}$= 0.576), and moderate training loads (p = 0.140). Furthermore, swimmers woke 5–6 days a week with fatigue, significantly more during high training loads, than low (p = 0.003, $\overset{\text{2}}{\underset{partial}{\eta }}$ = 0.446).

Objective sleep data represents 13 swimmers (100% compliance, no missing data), as one swimmer found the wrist-worn device too uncomfortable during sleep. Average Actigraph sleep data for both night-time sleep and napping over differing training loads can be found in Table 1. When viewing all night-time sleep data, average TST was 06:30 ± 0:51 hrs: mins, while average latency was 00:14 ± 00:09 hrs: mins. Sleep efficiency for all swimmers, was on average 81 ± 5%. When napping, swimmers slept an average of 2:00 ± 00:51 hrs: mins, with an average latency of 00:08 ± 00:04 hrs: mins.

**Table 1:** Average Actigraph sleep data over differing training loads.

	Training Load	Latency (min)	Efficiency (%)	TTIB (min)	TST (min)	WASO	No. of Awakenings	Average Time Awake (mins)
Night-time	Low	17 ± 12	81 ± 6	553 ± 28	445 ± 35	90 ± 27	30 ± 5	3 ± 0
	Mod	12 ± 6	82 ± 4	451 ± 48 *	366 ± 30 *	72 ± 24	22 ± 7 *	3 ± 0
	High	12 ± 5	81 ± 5	449 ± 49 ◊	363 ± 38 ◊	74 ± 24	25 ± 6	3 ± 1
Napping	Low	10 ± 3	70 ± 8	170 ± 71	115 ± 37	45 ± 37	7 ± 4	6 ± 4
	Mod	8 ± 5	80 ± 6	152 ± 58	122 ± 46	21 ± 15	7 ± 4	3 ± 1 *
	High	4 ± 3 *◊*	83 ± 10	150 ± 67	126 ± 66	20 ± 13	6 ± 2	3 ± 2 *◊*

Note*.* Night-time and napping data for all swimmers (*n* = 13), during low, moderate and high intensity training (loads derived by coaches). *Denotes a significant difference between low and moderate training loads, for the variables marked. ◊Denotes a significant difference between low and high training loads, for the variables marked. Statistical significance was accepted at p ≤ 0.05. TTIB, total time in bed; TST, total sleep time; WASO, wakes after sleep onset.

For night-time sleep periods that preceded an early morning training day, swimmers on average went to bed at 22:32 ± 00:54 hrs: mins, woke up at 04:56 ± 00:49 hrs: mins, and obtained 05:00 ± 00:58 hrs: mins of TST. For sleep periods where there was no early morning training, swimmers on average went to bed at 22:55 ± 03:07 hrs: mins, woke up at 08:53 ± 01:12 hrs: mins, and obtained 07:42 ± 01:07 hrs: mins of TST. Swimmers had significantly less TTIB (p = 0.001) and TST (p = 0.001) on nights before early morning training, compared to nights with no morning training. Additionally, significantly less WASO (63 vs. 99; p = 0.001) and number of awakenings (20 vs. 32; p = 0.001), were observed on nights before early morning training. No differences were found between nights preceding and not preceding early morning training for sleep efficiency (79% vs. 80%; p = 0.755), average time awake (3 mins vs. 3 mins; p = 0.193), or sleep latency (18 mins vs. 14 mins; p = 0.122). Objective sleep data leading into competition can be seen in Table 2. Additionally, average sleep score before competition was reported as 6.2 (out of 10), compared to 7.2 during mid-season training.

**Table 2:** Average ActiGraph sleep data two weeks prior to major competition.

Weeks Before Competition	Latency (min)	Efficiency (%)	TTIB (min)	TST (min)	WASO (min)	Number of Awakenings	Average Time Awake (min)
Two	11 ± 12	82 ± 7	470 ± 118	382 ± 94	77 ± 41	24 ± 11	3 ± 1
One	19 ± 20	80 ± 8	552 ± 89 *	439 ± 84 *	93 ± 40	30 ± 9 *	3 ± 1

Note. *Denotes a significant difference between one and two weeks before competition, for the variables marked (*n* = 13). Statistical significance was accepted at p ≤ 0.05. TTIB, total time in bed; TST, total sleep time; WASO, wakes after sleep onset.

## Discussion

Main findings of the current study were the significant relationships found between self-perceived sleep, incidence of URS, symptom severity, and relative salivary IgA. The more days a swimmer awoke feeling fatigued, incidence of URS and severity increased, and relative salivary IgA declined below an individual’s healthy average; although the direction of causality remains unclear. Additionally, the more times a swimmer felt they met the NR of sleep, both incidence of URS and symptom severity score increased, suggesting swimmers may actively try to sleep more for enhanced recovery. Further findings showed that average sleep quantity obtained by elite British swimmers was less than the NR, plus was significantly reduced during high training loads and on early morning training days. Average sleep efficiency was 81% throughout the study period. Furthermore, competition influenced sleep; swimmers’ TST significantly increased from two weeks to one week before competition, however, total number of awakenings increased leading into competition.

Interestingly, swimmers who reported waking feeling fatigued on more days showed higher incidence and severity of URS, with reductions in relative salivary IgA below their individual healthy average. This complements previous work that reported that inadequate sleep could lower immunity and increase chance of illness [23]. That said, it is also possible that waking with fatigue reflects the effect of illness, rather than being solely a cause. Another significant positive association was found between the perception of days meeting the sleep NR, with both URS incidence and severity. At first glance, this is contrary to other literature; where less than seven hours’ sleep meant that individuals were 2.94 times more likely to develop a cold compared to those with eight hours or more [3]. Interestingly however, swimmers reported sleeping the NR significantly more frequently during moderate and high intensity training loads, when significantly higher incidence, duration and severity of URS was also reported [20]. Therefore, findings may instead reflect swimmers’ perception of an increased requirement for rest and recovery during high training periods and illness, perhaps combined with the associated feelings of fatigue discussed. Self-reported sleep, in addition to the use of ActiGraphs which has been previously validated against Polysomnography (PSG) (the “gold standard”) [24], was a major strength of the current study.

Objective night-time sleep data showed swimmers slept an average of 06:30 hrs: mins, which was similar to previous research in swimmers [10,11]. Findings supported a recent review of 54 studies that found athletes were often unable to achieve ≥7 hours of TST [1]. Possible explanations for this have been suggested to be the result of increased training load or changes in training schedule [25]. In the current study, both TTIB and TST were significantly less during moderate and high training load, compared to low, suggesting that a higher training load may negatively impact sleep quantity. Current findings were supported by previous literature in elite synchronised swimmers [26], cyclists [14,27], gymnasts [16], and swimmers [28]. On nights prior to early morning training days, swimmers spent less TTIB and obtained significantly less TST compared with nights with no early morning training. Swimmers got out of bed just under 4 h earlier on early morning training days (04:56 vs. 08:53 hrs: mins) and interestingly did not attempt to compensate for having to wake earlier by going to bed earlier (22:32 vs. 22:52 hrs: mins). Current swimmers had early morning training up to five times a week and thus is common practice; however, this level of sleep restriction can have a major effect on training and subsequent performance. Current findings agreed with other work [9] and reiterate that early morning training restricts sleep to a level far below the NR. Inconsistent sleep-wake times are thought to disrupt circadian rhythms, negatively impacting hormonal and metabolic homeostasis [29], and should therefore be avoided where possible. A recent review found an overwhelming body of evidence showing that irregular sleep schedules were not associated with improved outcomes for health and performance in any study they examined [30]. In this context, scheduling training sessions in alignment with an individual’s circadian preference, rather than imposing fixed early-morning start times, may be beneficial; however, fixed pool availability and shared training sessions limit the scope for individualisation for sleep. Consequently, targeted support for individuals most vulnerable to early training demands may be a more practical solution for coaching staff.

Sleep efficiency of ≥ 85% was said to be a strong indicator of good sleep quality [12], however, swimmers in the current study did not meet this (81%). These findings agree with a recent review [1], however were slightly lower than what has been found elsewhere in elite swimmers (82%) [10,11]. Sleep efficiency findings in the current study could have been driven by TTIB and sleep latency. That said, swimmers met good sleep latency recommendations of ≤15 minutes [12] (14 minutes) and exceeded those reported elsewhere (35–40 minutes) [14]. However, total WASO in the current study (79 minutes) was much higher than that shown in other studies ranging from 14–65 minutes [10,11,14,31]. Researchers examining a cohort of 47 Olympic athletes found WASO to be similar to the current study [8], and proposed that wrist-worn actigraphy may potentially be affected by those with larger amounts of movement [8,32]. Overall, greater sleep fragmentation has been reported in athletes [33], which could be the case in the present study; and therefore, may explain the high WASO and inadequate sleep efficiency.

In the current study, TTIB and TST significantly increased from two to one week before competition. Additionally, a lower percentage of swimmers woke feeling fatigued in the week leading into competition, with “1-2 times” being the most reported (52%). These findings may be accountable to taper, reduction in training load and/or swimmers actively trying to rest when leading into major competition. Total number of awakenings, however, were significantly higher one week leading into competition, compared to two. Although speculative, nervousness and thoughts about the competition could be causing the increase in number of awakenings [17]. That said, it should be considered that the observed increase in TTIB could simply have provided greater opportunity for awakenings. When examining self-reported sleep quality though, average sleep score was lower before competition (6.2) than mid-season training (7.2). Previously, two large cohort studies found 64–66% of athletes experienced poor sleep leading into competition (n = 915) [17,18], supporting current findings and further adding support to this notion.

The clinical implication of these findings is that elite sport may benefit from identifying athletes at risk of sleep inadequacy and associated URS, as demonstrated in the current study. Given that changes in sleep can alter immune markers [34], the importance of meeting the NR of sleep and minimising illness and fatigue is evident, supporting the use of sleep monitoring methods. The requirement to take an individualised approach to managing swimmers’ training and competition schedules, whilst using a physiological biomarker and subjective data to inform decisions has been highlighted [20]. Whilst causality cannot be established, perceptions of waking with fatigue may serve as a practical indicator of periods associated with reduced mucosal immunity and increased URS, further emphasising the relationship between sleep and immunity. Accordingly, the implementation of strategies to improve sleep appears warranted. Unsurprisingly, simple sleep hygiene methods (eye masks, afternoon napping, avoiding screens, etc.) over just ten consecutive days was proven effective in improving sleep in 14 Olympic swimmers [11]. Furthermore, a recent review found 1–2 hours of catch-up sleep potentially beneficial when sleep duration was inadequate, promoting napping for the current cohort [30]. Once identifying those at increased risk, sleep education could help improve sleep and reduce subsequent illness burden and fatigue in athletes.

A limitation of the current study includes having a small sample size [20]. As previously discussed, meeting participant recommendations is often unrealistic when recruiting elite athletes [35]. Whilst our previous work addressed individual differences in URS, insufficient sleep data limited the ability to examine individual variability of sleep. With the significant relationships found here, future research could better explore individual sleep patterns alongside causal direction for illness, to inform training and recovery strategies. Furthermore, despite addressing sleep as a potential risk factor for URS, it must be mentioned that other factors exist which were not accounted for, such as nutrition, stress and/or living arrangements (shared accommodation). Regarding self-reported sleep data, the weekly questionnaire used was adapted and therefore, not formally validated. Also, the questionnaire was completed weekly, increasing the potential for recall bias. That said, we believe that collecting self-reported sleep data alongside objective actigraphy data, was a strength of the current study. Lastly, although health screening was conducted, sleep disorders were not screened for and therefore, should be acknowledged as a limitation.

## Conclusion

The current study presents a well-controlled, longitudinal observation of sleep, alongside mucosal monitoring and self-reported URS, in elite international swimmers. It seems that the perception of waking feeling fatigued was associated with URS and lowered relative salivary IgA in the current study. Whilst this may be a predictive indicator, further validation is required. These findings should promote both the importance of individual sleep monitoring and the beneficial effect it could have on health and subsequent training and performance. Findings additionally strengthen the notion that swimmers regularly sleep below the NR and that high training loads and early morning training further negatively impact sleep duration. The importance of educating athletes on effective sleep hygiene practices is highlighted by this study, together with considerations as to the appropriateness of training schedules which force large shifts in circadian rhythm. The requirement to take an individualised approach is evident; coaches should consider monitoring self-perceived sleep, fatigue, and URS alongside measurement of salivary IgA[21], to correctly manage each individual swimmers’ training and competition schedules. This could have a positive influence in elite sport, by promoting the identification of individual differences to allow unique tailoring of training to improve subsequent performance and athlete wellbeing.

## Supporting information

S1 File Weekly Questionnaire. (PDF)
