# Correction: Sleep inadequacy and the relationship with mucosal immunity and upper respiratory symptoms in elite swimmers: A longitudinal study leading into the Commonwealth Games

**DOI:** 10.1371/journal.pone.0359216

**Published:** 2026-09-24

**Authors:** Lauren H. Baker, Terun Desai, Jonathan Sinclair, Amy V. Wells

The images for Figs 1 and 3 are incorrectly switched. The image that appears as Fig 1 should be Fig 3, and the image that appears as Fig 3 should be Fig 1. The figure captions appear in the correct order. The authors have provided a corrected version of the figures here.

**Fig 1:**
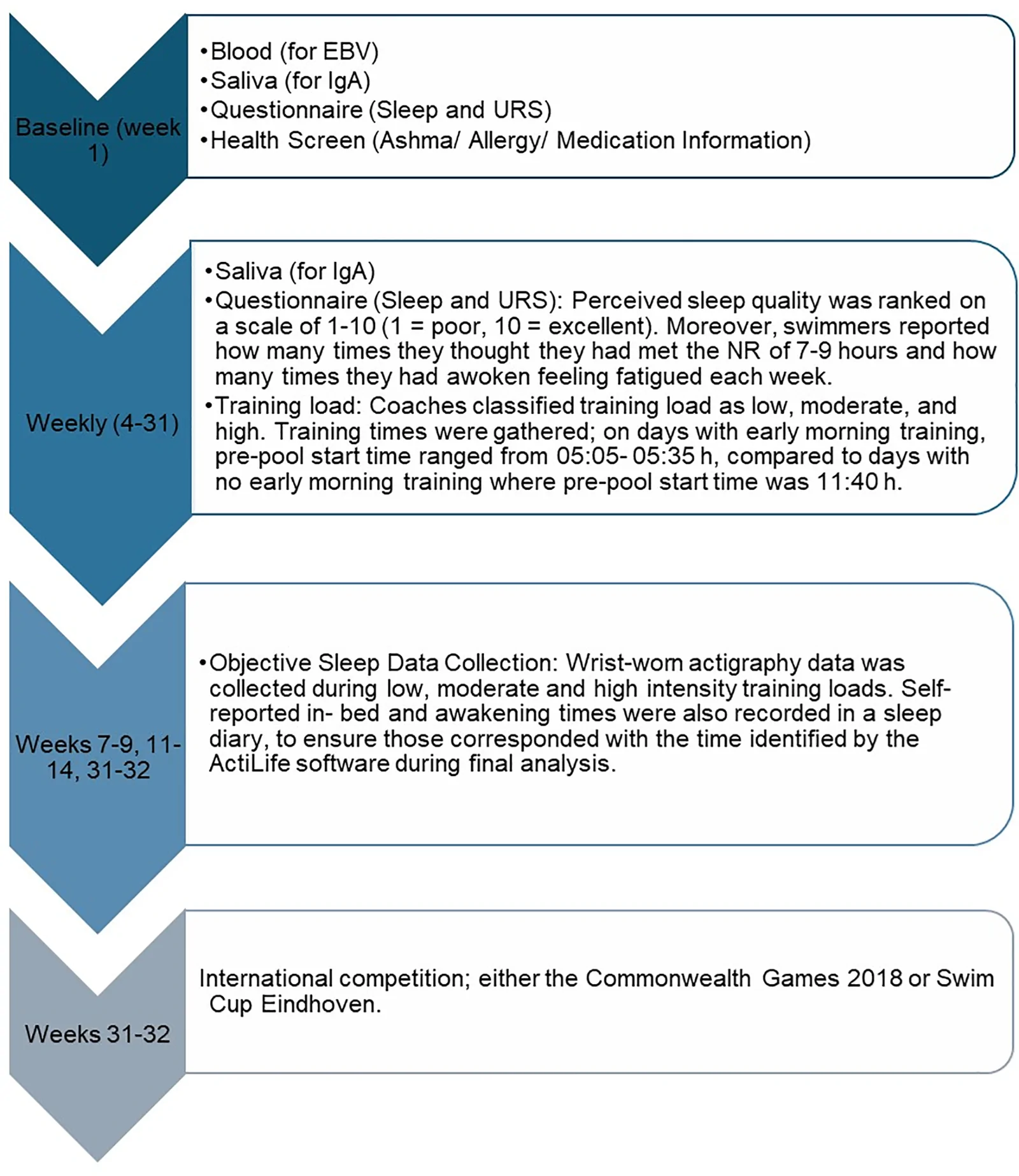
Study design flow diagram.

**Fig 3:**
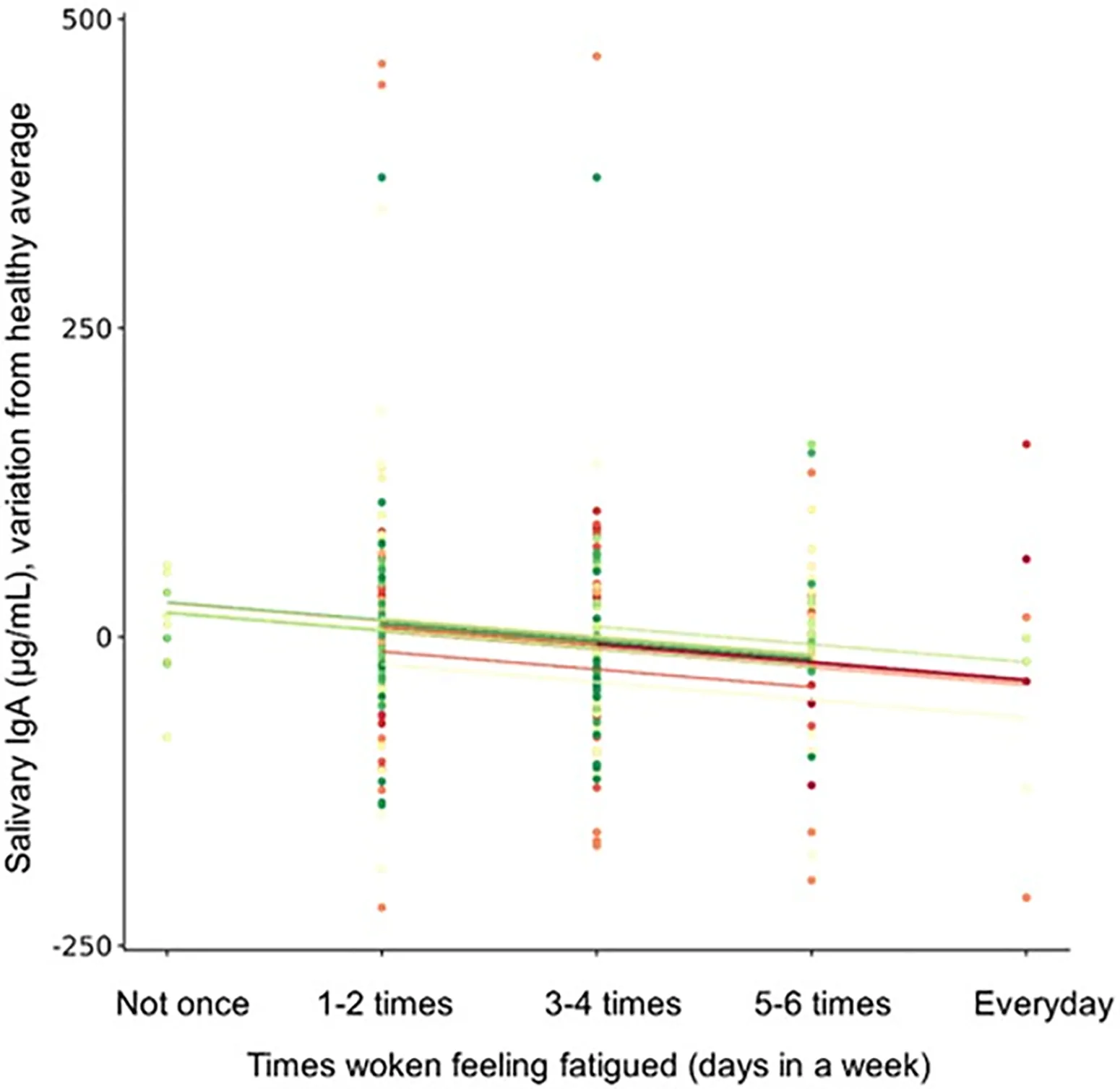
Reported times woken feeling fatigued alongside salivary IgA. A weak negative relationship was found with swimmers waking fatigued and relative salivary IgA (the variation from their healthy average) (rrm (304) = −0.12, 95% CI [−0.229, −0.008], p = 0.035).

## References

[1] Baker LH, Desai T, Sinclair J, Wells AV. Sleep inadequacy and the relationship with mucosal immunity and upper respiratory symptoms in elite swimmers: A longitudinal study leading into the Commonwealth Games. PLoS One. 2026;21(4):e0346138. doi: 10.1371/journal.pone.0346138 41926426 PMC13046147

