# Supplementary material for: Sleep inadequacy and the relationship with mucosal immunity and upper respiratory symptoms in elite swimmers: A longitudinal study leading into the Commonwealth Games
Source: PLoS One. 2026 Apr 2;21(4):e0346138. doi: 10.1371/journal.pone.0346138 (PMC13046147; doi:10.1371/journal.pone.0346138)
Supplement: S1 File — (PDF) [file pone.0346138.s001.pdf]

S1 File. Weekly Questionnaire

Name: ___________________________________

Date: ___________________________________

Week: ___________________________________

Step 1: Circle your answers for sleep, training, injury and illness sections.
Step 2: Please state what injury/illness, whether you have seen a doctor and what medication you received.
Step 3: To the best of your ability, please number each illness appropriately for the past week (1- Minimal, training was normal, 2- Moderate, training was modified, 3- Severe, did not train). If you had no illness, please leave them blank.

Sleep:

This week, how often have you met the 7-9 hours of sleep (per day) recommendation?

Everyday	5-6 times a week	3-4 times a week	1-2 times a week	Not once

How would you rate your quality of sleep, for this week? (1 poor- 10 good)

1	2	3	4	5	6	7	8	9	10

How often this week have you felt tired or fatigued, after waking up in the morning?

Everyday	5-6 times a week	3-4 times a week	1-2 times a week	Never

Training

In your opinion, what intensity was swim training this week? (1 low- 10 high)

1	2	3	4	5	6	7	8	9	10

In your opinion, what intensity was weight training this week? (1 low- 10 high)

1	2	3	4	5	6	7	8	9	10

Injury?

Yes	No
If yes, please state:	

Illness?

Yes	No
If yes, please state what:	
Did you see a doctor? Yes/No	Have you taken any medication? Yes/No<br>If yes, please state what:

	Thursday	Friday	Saturday	Sunday	Monday	Tuesday	TODAY
Upper Respiratory - blocked or runny nose, sore throat, sneezing							
Chest Infection - coughing, sputum, chest congestion, wheezing, high temperature							
Muscles and joints - aching or swollen (not related to injury)							
General fatigue - lethargy, tiredness							
Head - headache, migraine, dizziness, vision impairment, vertigo, glare/light intolerance							
Ears - ear ache, ringing in the ears, hearing loss							
Rashes - specify - localised (L) or widespread (W)							
Skin infections - sores, boils, deep abscesses, infected blisters, athlete's foot							
Gastrointestinal problems - nausea, vomiting, diarrhoea, abdominal pain, bloating, painful swallowing, loss of appetite							
Cardiovascular – feeling of fast heartbeat, shortness of breath, blackouts							
Eye irritation - itchiness, redness, sticky discharge, watery eyes							
Psychological - feeling depressed or anxious (not related to major event), poor sleeping pattern							
Urinary tract - increased frequency of passing urine, pain/burning, bleeding, menstrual irregularity							
Other (please specify):							
